# Asymptomatic Cardiac Metastases From Esophageal Cancer: A Case Report Of Ante-mortem Detection And Literature Review

**DOI:** 10.7759/cureus.6387

**Published:** 2019-12-15

**Authors:** Carlo Signorelli, Amedeo Pergolini, Giordano Zampi, Andrea Vallone, Enzo Maria Ruggeri

**Affiliations:** 1 Oncology, Belcolle Hospital, Viterbo, ITA; 2 Cardiology, San Camillo-Forlanini Hospital, Rome, ITA; 3 Cardiology, Belcolle Hospital, Viterbo, ITA; 4 Radiology, San Camillo-Forlanini Hospital, Rome, ITA

**Keywords:** cardiac metastases, esophageal cancer, ante-mortem detection, cancer case report, cardiac imaging

## Abstract

Metastatic spread to the heart from neoplasms is very rare, often silent and rarely gains clinical attention. Usually, it correlates with widespread metastatic disease and is suggestive of a poor prognosis. Most cardiac metastases (CM) are detected following post-mortem studies with only a handful reported antemortemly. Here, we report a case of an asymptomatic cardiac metastasis from esophageal carcinoma and a review of the literature. In late July 2014, a 73-year-old woman diagnosed with locally advanced esophageal squamous cell carcinoma was admitted to our institution. Cardiothoracic metastases were not detected at basal computed tomography (CT) scan. The patient was submitted to concurrent cisplatin and radiotherapy. Just before surgery, a CT scan revealed two metastases in the right ventricle and in the interventricular septum. Transthoracic echocardiography and an endomyocardial biopsy confirmed the diagnosis of squamous cell carcinoma from the esophageal origin. In February 2015, chemotherapy was started, but after two courses of gemcitabine, a pulmonary embolism and then a congestive heart failure caused death of the patient on April 2015. Reviewing the literature, 14 cases with an antemortem diagnosis of CM from esophageal cancer were reported. Our patient should be the fifteenth case with an uncommon presentation without symptoms or signs in the diagnosis. Our case highlights that patients should be evaluated using echocardiography for CM, even if asymptomatic.

## Introduction

Cardiac metastases (CM) are very rare, often silent and rarely gain clinical attention. As in the primary tumors, the most frequent symptoms are congestive heart failure, valvular heart disease, pericardial effusion, electrocardiographic changes, dysrhythmia, syncope, embolism, cardiomegaly and it may arise suddenly. However, signs are often overlooked. Usually, it correlates with widespread metastatic disease and is suggestive of a poor prognosis. Although cardiac dissemination of malignant disease is a common finding during an autopsy, it rarely produces clinical symptoms [1]. Most of CM are detected following post-mortem studies with only a handful reported antemortemly. Epidemiological information is predominantly based on autopsy studies with incidences ranging from 2.3%-18.3% of cancer patients and from 0.2%-6.5% in unselected autopsy series [2-3]. Metastatic spread of cancers to the heart is more common than primary cardiac tumors. CM from tumors arising from the esophagus and pericardial effusions caused by metastatic cancer are rare; squamous cell carcinoma causing these events is extremely uncommon. It may appear during the evolution of this neoplasm and the tumor spread to the heart is usually caused by direct invasion. Not infrequently, cardiac tumor invasion contributes to the mechanism of death. The most of cardiac tumors are metastases mainly from pleural mesothelioma, melanoma, lung adenocarcinoma, undifferentiated carcinomas, lung squamous cell carcinoma, breast carcinoma, ovarian carcinoma, lymphomyeloproliferative neoplasms, bronchioalveolar carcinoma, gastric carcinoma, renal carcinoma, and pancreatic carcinoma and occur more commonly on the right side than the left side of the heart [2]. Only tumors of the central nervous system have not been proven to develop CM. Right ventricular metastases from esophageal cancer are even rare, with few such cases reported in the literature to date, being focused more on imaging findings from cardiac echocardiography [4]. Compared to metastases to the myocardium, direct extension to pericardium or regional lymphatic invasion are a more frequent route for invading the heart [2]. Since most of CM appear in the terminal stage of cancer, the prognosis is grave and therapeutic options are limited to palliative treatment of symptoms and chemotherapy. Here, we report a case of ante-mortem detection of asymptomatic CM from esophageal carcinoma and a review of the literature.

## Case presentation

**Figure 1:**
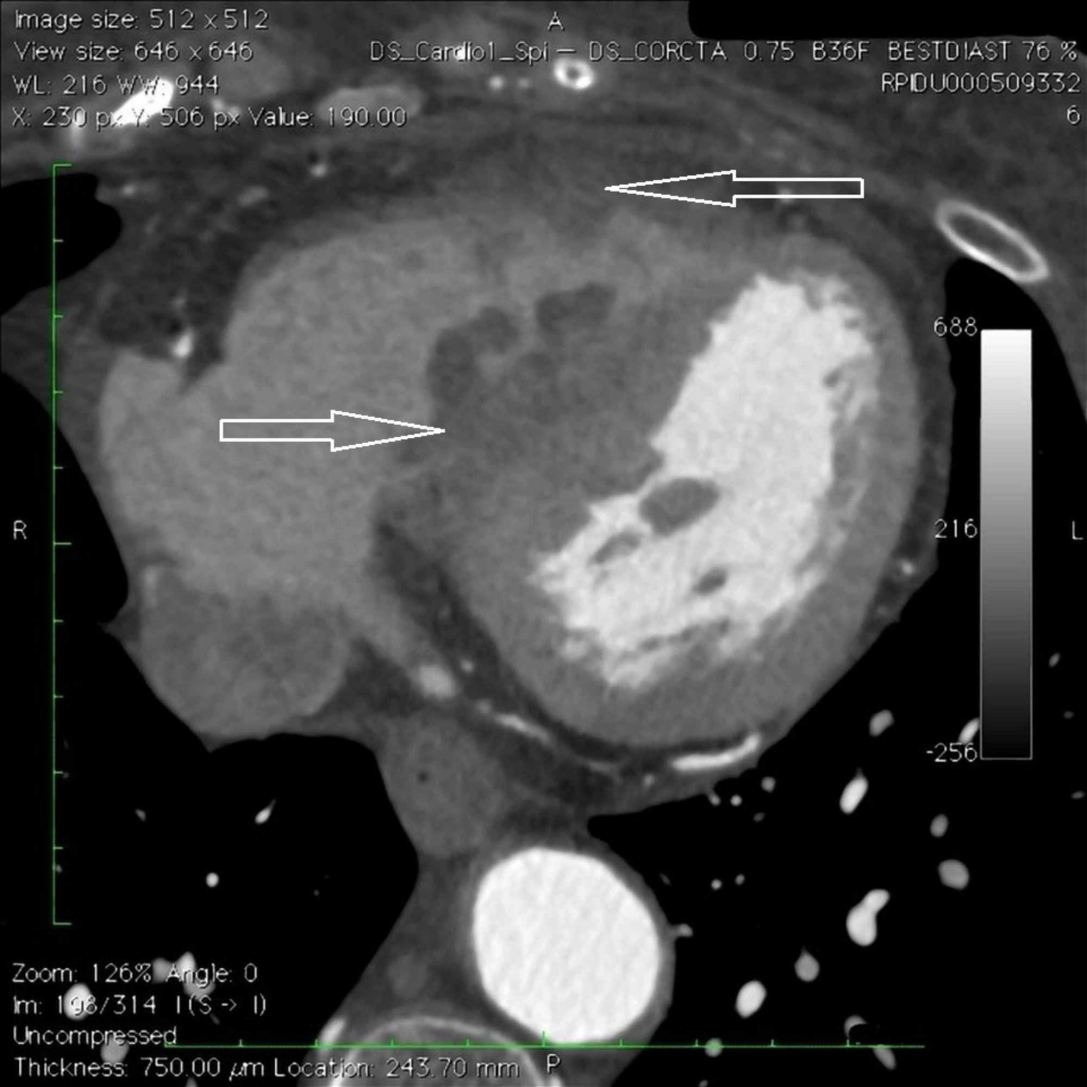
Computed tomography (CT) scan of the heart Axial plane contrast-enhanced.

**Figure 2:**
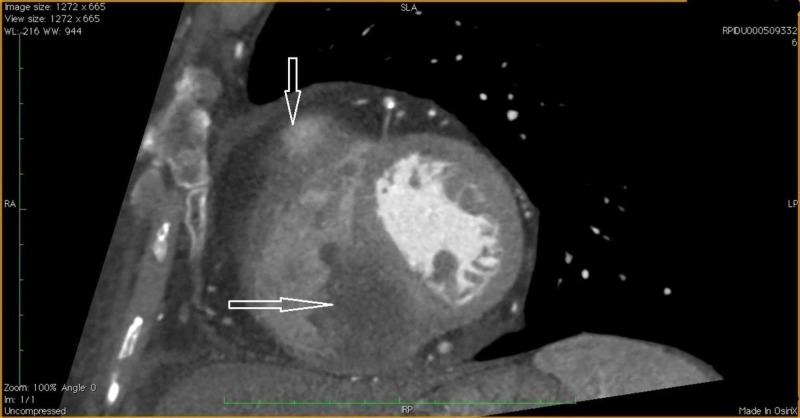
Computed tomography (CT) scan of the heart Multi planar reformation contrast-enhanced. Evidence of filling defect within the right ventricular cavity in correspondence with the interventricular septum.

In July 2014, a 73-year-old woman was diagnosed with locally advanced stenosing squamous cell carcinoma of the middle third of the esophagus (maximum diameter 40 mm) and was admitted to our institution. The patient had been suffering from chronic bronchitis secondary to tobacco consumption. Symptoms referred by the patient included dysphagia, weight loss, and right shoulder blade pain. Routine blood tests, including tumor markers and electrocardiogram (ECG) were normal. A computed tomography (CT) scan showed only the esophageal cancer with regional pathological lymph nodes. No basal evidence of cardiothoracic metastases. Transthoracic echocardiography revealed an ejection fraction of about 60%. The patient was planned to receive primary treatment with two courses of concurrent cisplatin (60 mg/mq) and radiotherapy (50 Gy totally with daily fractionation of 2 Gy). The patient had a clinical benefit. In November, just before undergoing surgery, a CT scan and an endoscopy showed a reduction of both the esophageal mass and lymph-nodes but revealed the presence of two suspected metastases with a maximum diameter of 50 mm, one in the right ventricle and one in the interventricular septum (Figures 1-2).

**Figure 3:**
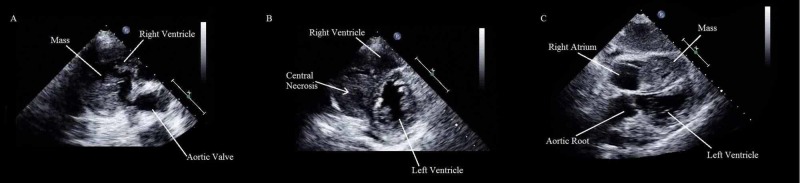
Transthoracic echocardiography A. Short-axis, great vessels off-axis view, showing a lobulated, cerebriform mass in the right ventricle near to the outflow tract. B. Short-axis, middle ventricle view, showing a mass occupying the right ventricle, adherent to the interventricular septum, with an anechoic central core. C. Subcostal view, showing a mass, with multiple anechoic foci, occupying a large amount of the right ventricle.

**Figure 4:**
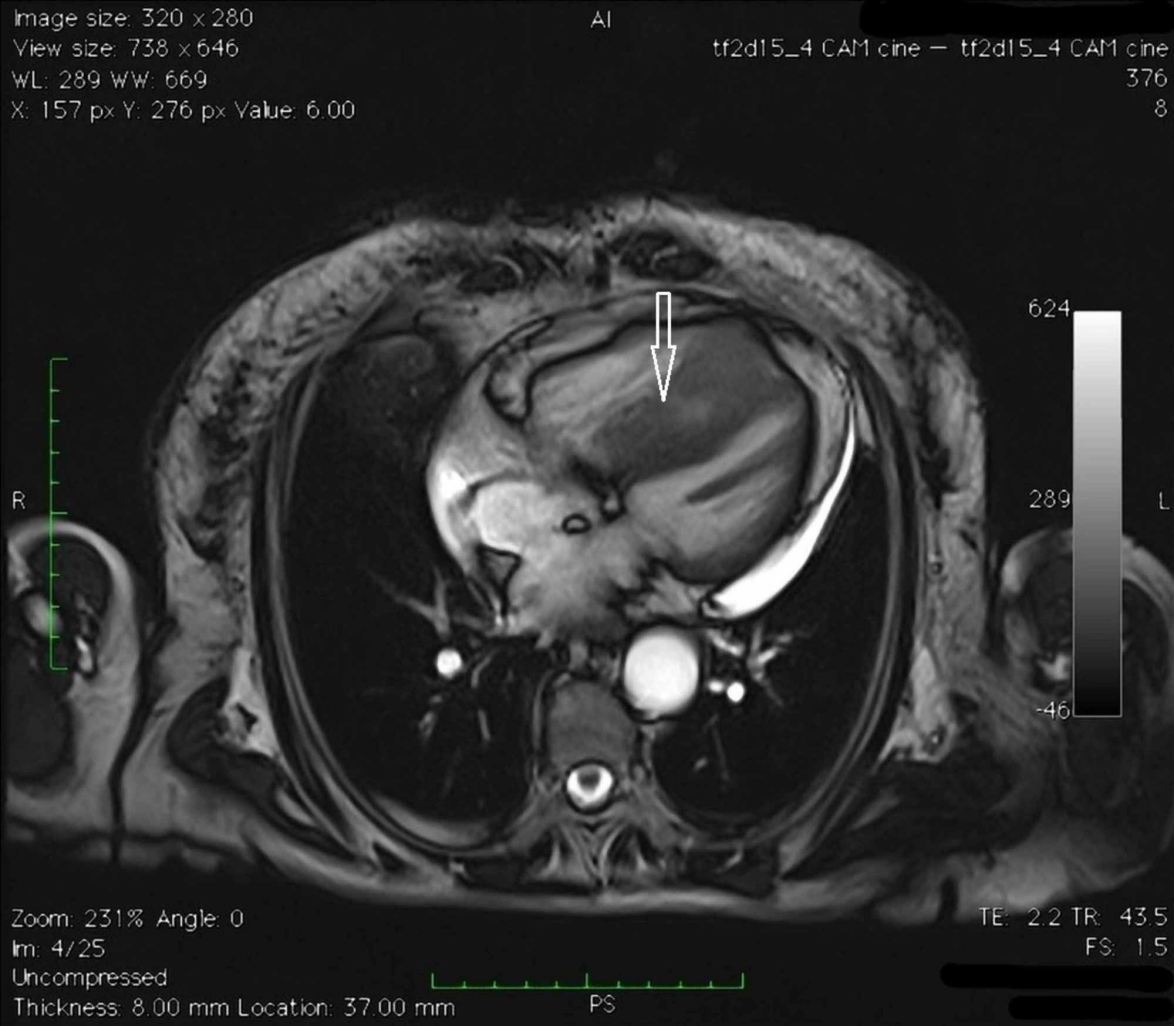
Cardiac magnetic resonance imaging (MRI) Four chambers cine MRI.

**Figure 5:**
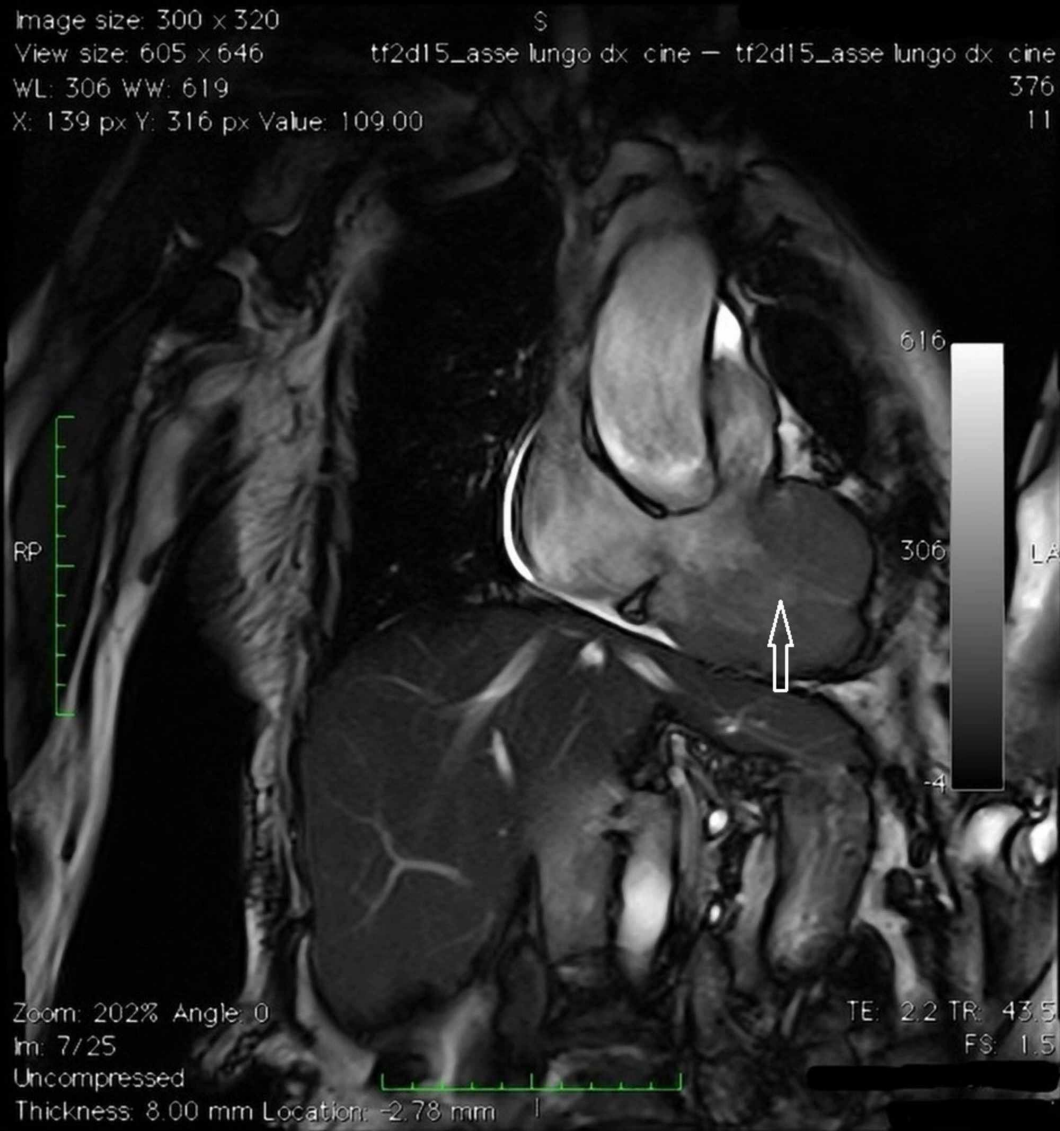
Cardiac magnetic resonance imaging (MRI) Four chambers cine MRI.

**Figure 6:**
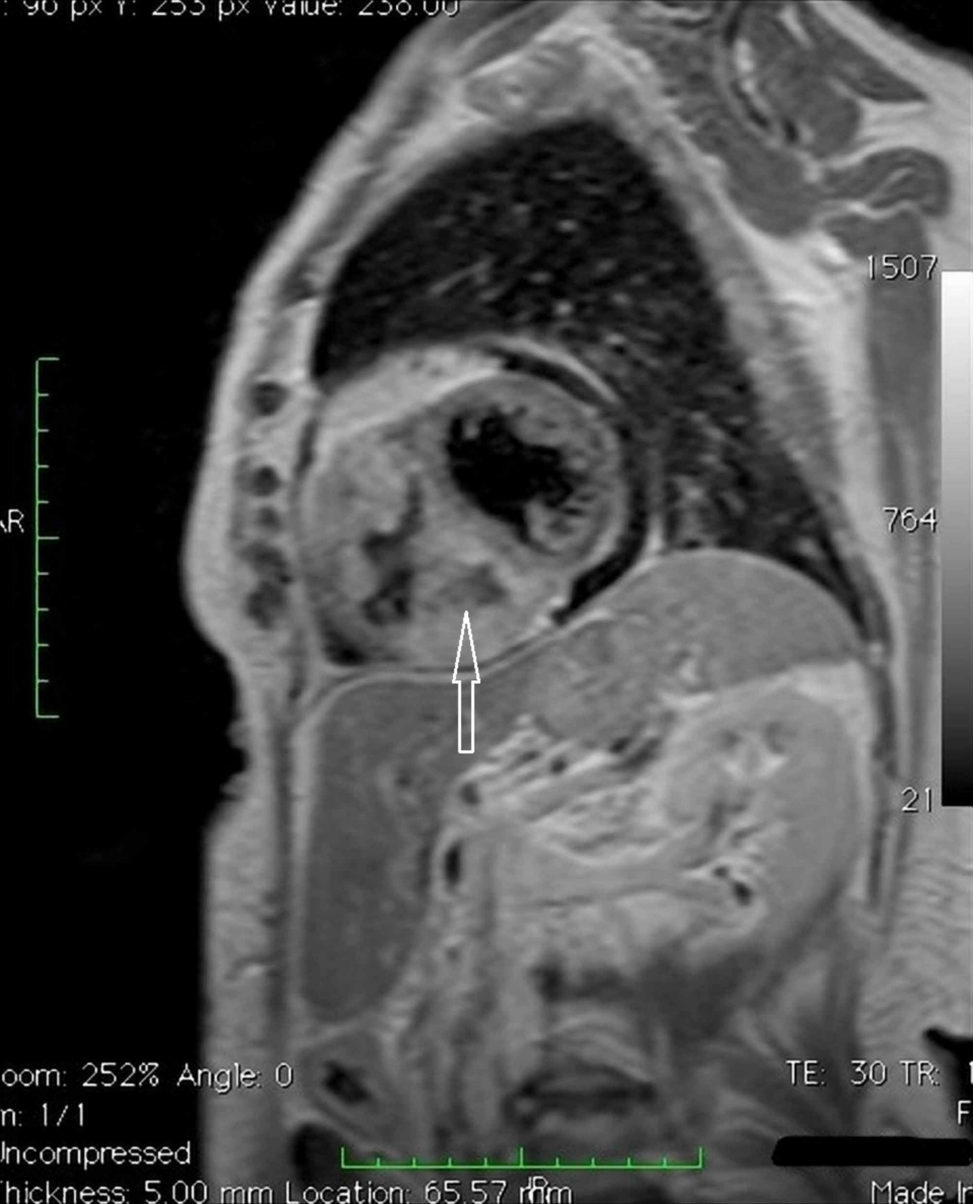
Cardiac magnetic resonance imaging (MRI) TSE_T1 2 chambers cine MRI targeted for the study of the right ventricle.

**Figure 7:**
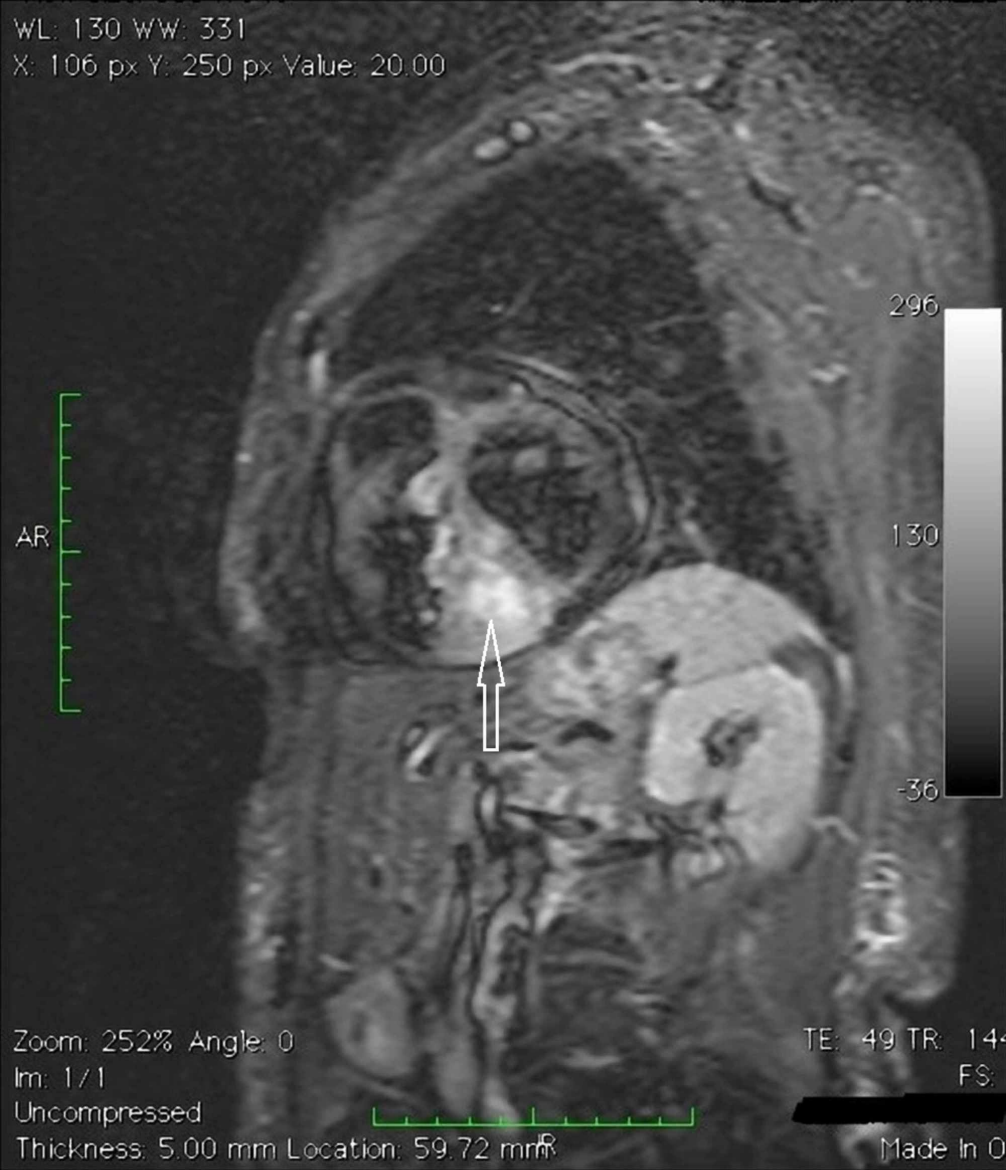
Cardiac magnetic resonance imaging (MRI) Turbo inversion recovery magnitude (TIRM) MRI. Confirmation of the expansive formation already observed on computed tomography-scan, another expansive lesion in the free wall of the right ventricle; evidence of necrosis within the mass.

The transthoracic echocardiography (as seen in Figure 3) and a magnetic resonance imaging (MRI) confirmed the two CM occupying the right ventricle near to the outflow tract and adherent to the interventricular septum (Figures 4-7).

**Figure 8:**
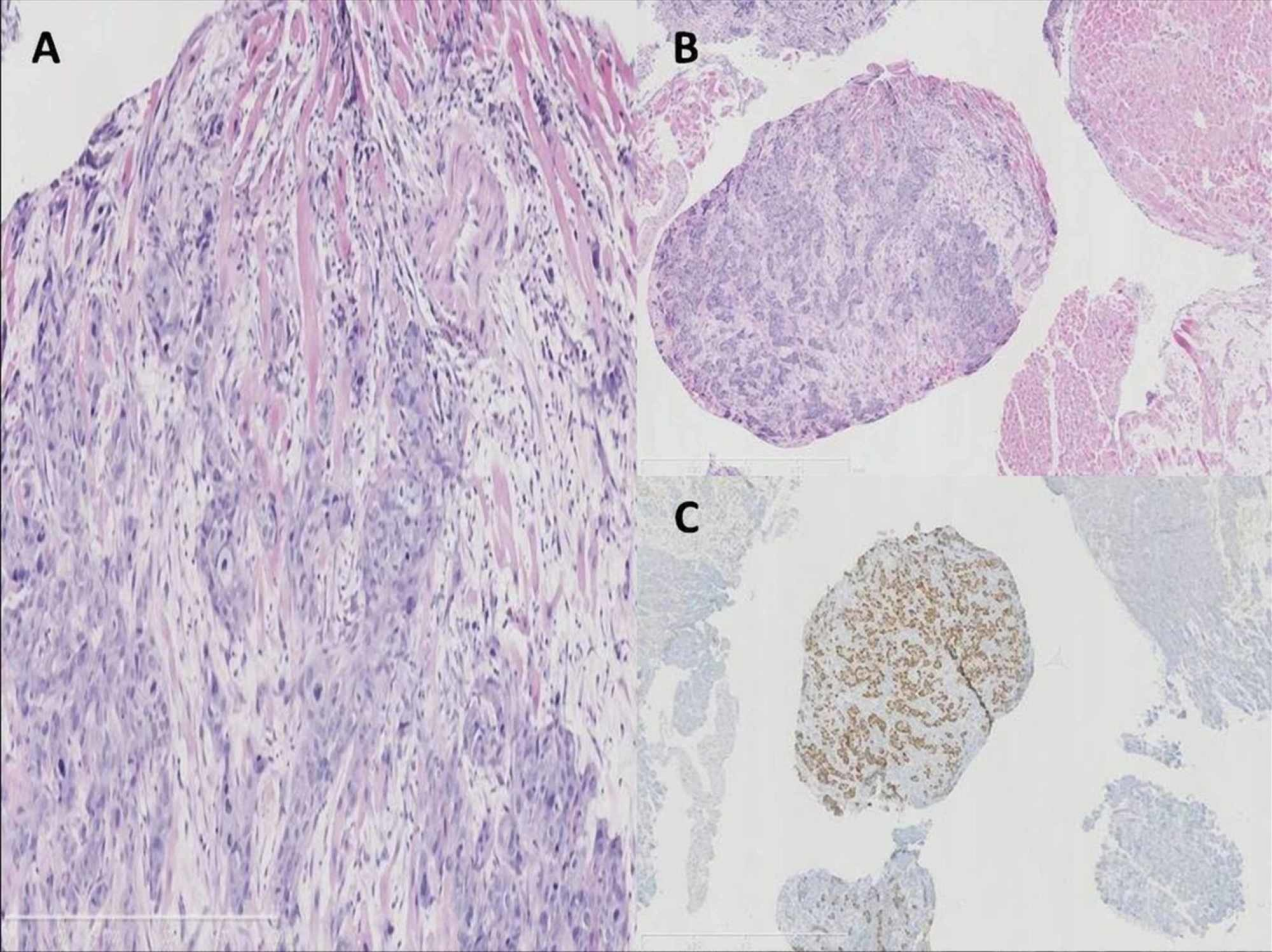
Photomicrograph of cardiac metastases biopsy A. Histopathological examination of the specimen shows that the tumor is a squamous cell carcinoma p63 immunostaining (hematoxylin and eosin stain; original magnification, ×100). B. Squamous cell carcinoma infiltrating the myocardial striated muscle, E-E. C. Squamous cell carcinoma p63 immunostaining. High magnification.

An endomyocardial biopsy revealed the diagnosis of squamous cell carcinoma from esophageal origin, p63+, cytokeratin 7 and 20-, CDX2- (Figure 8).

Both surgery and radiotherapy were not indicated as therapeutic options by a multidisciplinary oncology group. As the patient preference was to be treated, in February 2015 chemotherapy with gemcitabine was started, but after two courses a pulmonary embolism and then a congestive heart failure conducted her to death in April.

## Discussion

The presented case reports an ante-mortem diagnosis of CM from known esophageal cancer. As more of 90% of CM is silent, diagnosis is often difficult and mostly discovered at necropsy. If symptoms or signs are present, they are often overshadowed in the setting of widespread metastatic disease and the patient usually die from other metastatic lesions. Since the introduction of 2D-echocardiography, multislice CT, and MRI, CM have been more frequently diagnosed in vivo. Up until now, only 14 cases with an ante-mortem diagnosis and treatment of intracardiac metastases from esophageal cancer have been described in the literature (Table I).

**Table 1:** Literature review of cardiac metastases from esophageal cancer with an ante-mortem diagnosis

Author (year)	Age	Sex	Type	Primary Tx	Interval to cardiac metastasis	Recurrence diagnosis modality	Location	Symptoms or signs	Pathologic confirmation by	Recurrence Tx	Cause of death	Time to death from cardiac metastasis	OS
Karabay 2012 [5]	46	M	SCC	*	2M	echocardiogram	Myocardium LA, LV	Ventricular fibrillation	*	*	*	*	*
Youn 2002 [6]	65	M	SCC	eRT	simultaneous	Echocardiogram, MRI, coronary angiography	Endocardium RV	Heart murmur	Endomyocardial biopsy	eRT	*	*	*
Tayama 2011 [7]	48	M	SCC	none	simultaneous	echocardiogram	Epicardium RV	Cardiac tamponade, dyspnea	Open excisium	BSC	Rapid clinical deterioration	1M	1M
Cheng 2013 [8]	58	M	SCC	CCRT Surgery	*	Echocardiogram Chest CT scan	Myocardium LV	Dyspnea, Chest tightness mimicking myocardial infarction	*	CTx	*	*	*
Consoli 2011 [9]	67	M	SCC	CCRT	11M	Echocardiogram, MRI	Endocardium, pericardial effusion LV, RV	Dyspnea, palpitations, cardiac arrhythmia, hypotension	*	CTx	Neoplastic effusion	6M	16M
Oliveira 2012 [10]	81	M	SCC	Surgery	9M	Echocardiogram, CT scan	Myocardium, pericardium RV	Acute stroke	CT-guided needle aspiration biopsy		pneumonia	1M	10M
Basaran 2013 [11]	80	F	SCC	surgery	12M	echocardiogram	Myocardium, LA, LV	Ventricular tachycardia	*	BSC	RHF	0M	12M
Maeda 2009 [12]	62	F	SCC	surgery	6M	CT scan echocardiogram	Myocardium LV	none	*	CTx	cancer	11M	17M
Moulin-Romsee 2007 [13]	47	F	SCC	CCRT Surgery	*	FDG-PET/TC	* RA	none	*	BSC	cancer	*	*
Al-Mamgani 2007 [14]	60	M	*	CCRT	11M	CT scan ecocardiography	Myocardium RV	Chest pain	*	eRT	pneumonia	2M	13M
Nakamura 2005 [15] ^(12)^	53	F	SCC	*	simultaneous	CT scan ecocardiography	* Ball tumor in RA	Cardiac murmur Dyspnea on effort	Open excision	Surgery CCRT	cancer	5M	5M
Chello 1993 [16]	75	M	melanoma	Medical therapy	simultaneous	CT scan ecocardiography	* LA	Dysphagia Weight loss Pericardial effusion	experimental protocol with 99mTc labelled antimelanoma monoclonal Ab	BSC	*	*	*
Abbate 2015 [17]	49	F	SCC	CTx CCRT Surgery	20M	CT scan MRI	* LV	none	surgical debulking	CTx	ischemic stroke	12M	44M
Trocino 2015 [18]	48	F	SCC	Surgery	36M	CT scan Echocardiography MRI	Myocardium LV	none	myocardial biopsy	CTx	massive cardioembolic cerebral stroke	10M	46M
Our case 2019	73	F	SCC	CCRT	5M	Echocardiography, MRI, CT scan, coronary angiography	Endomyocardium RV	none	Endomyocardial biopsy	CTx	RHF	6M	10M
*no available data; CCRT, concurrent chemoradiotherapy; CT, computed tomography; CTx, chemotherapy; eRT, external radiotherapy; M, months; MRI, magnetic resonance imaging; RHF, right heart failure; SCC, squamous cell carcinoma; Tx, therapy.													

The majority of these cases were not single metastases. Main characteristics of these cases were: median age 59.9 years (the incidence of CM reported from autopsies is variable ranging from 2.3% to 18.3%, predominantly in the sixth and seventh decade of life); prevalence of male (57.1%) (although the frequency of primary tumours was found to be different for men and women, 46% in men and 31.7% in women, no differences were detected in the incidence of CM from post-mortem examinations); the predominant histologic type was squamous cell carcinoma (92.3%); median time to the onset of this kind of metastases: 8.9 months; the method of choice to detect recurrence was echocardiography in 78.5% of cases; the right side of the heart was involved in the same way as the left (50%); the structure primarly affected was myocardium (70%) (unlike the cases diagnosed post-mortem in which the most common site was the epicardium); the main treatment with palliative purposes was surgery (36.3%), followed by concurrent chemo-radiation, chemo-radiation before surgery (18.1%), best supportive care, radiotherapy and chemotherapy followed by concurrent chemo-radiotherapy before surgery (9%); the median time to death from Cardiac metastases was 5.3 months with an overall survival of approximately 18.2 months [5-18]. Survival is determined by the inherent aggressiveness of the cancer manifested by tumor size, grade and distant metastasis at presentation. To date, our patient is the fifteenth case in the literature, with an uncommon presentation without symptoms or signs with a median time to death from cardiac recurrence and an overall survival respectively of six and 10 months.

A variety of diagnostic imaging and hemodynamic techniques have been applied in the diagnostic process of all cases published, including echocardiography, CT, MRI, and even right heart catheterization. The method of choice to detect CM was two-dimensional echocardiography, the non-invasive imaging technique enabling clarification of the location, size, shape, number and mobility of the masses but it is usually limited in tissue characterization. Transesophageal echocardiography can give more information than the approach transthoracic and in the case of patients with specific physical habits, overcomes the disadvantage of poor acoustic window. However, echocardiography is usually limited in tissue characterization. Definite diagnosis requires pathological examination of tumor tissue. Electrocardiography is not specific. Localized and prolonged ST-segment elevation without Q waves appears to be pathognomonic for myocardial tumor invasion. Cardiac MRI could be the diagnostic tool of choice in the assessment of patients with esophageal cancer when CM are suspected; it offers improved resolution, a larger field of view and superior soft-tissue contrast, it is not invasive and does not require ionizing radiation or exposure to nephrotoxic contrast agents. Although there is overlap of the MRI characteristics of several cardiac masses, MRI is a reliable tool to identify necrosis, extra-cardiac spread, pericardial effusion and to exclude lipomas, fibromas and hemangiomas as well as thrombus or lipomatous hypertrophy. However, its inability to evaluate calcification constitutes an important limitation of MRI. Multidetector computed tomography is useful for the evaluation of calcification and fat content of a cardiac mass [19]. In descending order of frequency, epicardium (75.5%), followed by myocardium (38.2%) and endocardium (15.5%) are involved [20]. In our patient, hematogenous spread was a possible route for cardiac involvement, since the tumor was located on the ventricular septum and protruded into the right ventricular cavity. CM are usually bilateral, small and multiple; however, single large lesions are also observed. Valvular metastatic deposits, intracavitary or endocardial such as the one described in our case, occur in less than 6% of cases. Tachyarrhythmia is found often in these patients and may lead to syncope. Coronary occlusion or compression from tumor masses can lead to myocardial infarction, heart failure, and death. A typical clinical pattern of cardiac disease progression is characterized by a worsening performance status, exacerbation of cardiac symptoms, including tamponade, arrhythmia, obstruction, or dilated cardiomyopathy, representing one of the terminal events as in the case of the reported patient. CM are usually diagnosed in the setting of generalized carcinomatosis. At this stage, treatment must be with palliative intention to improve the quality of life, while in rare cases with a long life expectancy and a good performance status, when the heart is the only site of metastasis, it could be with curative intent.

Current treatment options do little to encourage a positive outlook and are particularly limited in cases of CM. Because cardiac tissue involvement is frequently diffuse, the usefulness of chemotherapy or surgery is questionable. Chemotherapy is recommended in chemosensitive primary tumors. Surgery is the treatment of choice in highly selected patients with a long life expectancy and a good performance status and it is only indicated in exceptional cases of solitary intracavitary heart metastases leading to obliteration of the cardiac chambers or valve obstruction. Surgery offers the best chance of prolonged survival. Because patients suitable for surgery generally have a better prognosis than inoperable patients, postoperative chemotherapy and/or radiotherapy should be given to reduce the chance of local recurrence. In such patients, it is important to keep the treatment time as short as possible. Radiotherapy should be attempted to relieve symptoms, to produce local control, and to stabilize hemodynamic disturbances. Short-course radiotherapy may be suitable to achieve these objectives. Radiotherapy may lead to fibrosis of the lung or of the myocardium, and thus to disorders of the conduction system and also to pericarditis. Whatever the treatment selected, the clinical evolution is disappointing, and patients with heart metastases die within a year of the diagnosis. This poor prognosis makes it important to define the most appropriate therapeutic strategy may be taking the approach to surgery in cases where the latter is possible.

(Abstract: Signorelli C, Chilelli MG, Pergolini A, Zampi G, Ruggeri EM. Ante Mortem Diagnosis of Intracardiac Metastases from Known Esophageal Cancer: A Case Report and Literature Review. 17th National Congress of Medical Oncology; October 23-25, 2015). https://academic.oup.com/annonc/article/26/suppl_6/vi/216104

## Conclusions

CM are unfavorable prognostic factors of survival and therapies are mostly palliative in order to ameliorate symptoms since cardiac dissemination mostly occurs in the setting of widespread metastatic disease. The therapeutic efficacy of an antineoplastic treatment must be balanced with its side effects and quality of life. The decision should involve a multi-disciplinary team. As heart metastases are often incurable, surgery is not an optimal option but in some patients can be useful for the relief of symptoms. Systemic chemotherapy is usually the most beneficial treatment. In patients who have known metastatic neoplasms and who present with cardiac manifestations, the clinician should be alert to the possibility of CM. However, treatment of all the metastatic cardiac tumors provides only palliation. Echocardiography performed periodically in patients with esophageal carcinoma could permit recognition of CM before the occurrence of overt cardiac signs leading to a chance for an aggressive surgical therapy. Accumulation of reports about treating cases of CM may help to clarify the natural course of this kind of lesions and how to treat them. After our review, we suggest that patients with a high risk of recurrence should be carefully evaluated using echocardiography for CM, even without symptoms. With the advent of modern diagnostic tools, better chemotherapy regimens, and better radiation techniques, and with the implementation of better perioperative care, patients with cancer have a somewhat improved survival. With increased longevity, a trend of increased incidence of CM has been shown in different studies. Because of the above-mentioned factors, together with the widespread use of modern imaging techniques, an increase in the frequency of the ante-mortem diagnosis of heart metastases is to be expected in the near future.
